# IgG4-Related Nasal Pseudotumor

**DOI:** 10.1155/2015/749890

**Published:** 2015-02-12

**Authors:** L. K. Døsen, P. Jebsen, B. Dingsør, R. Haye

**Affiliations:** ^1^Department of Otorhinolaryngology, Lovisenberg Diakonale Hospital, 0440 Oslo, Norway; ^2^Department of Pathology, Oslo University Hospital, Rikshospitalet, 0372 Oslo, Norway; ^3^Department of Otorhinolaryngology, Telemark Hospital, 3710 Skien, Norway

## Abstract

IgG4-related disease is recognized as one form of autoimmune pancreatitis. During the last ten years, it has also been described in several other organs. We present two patients with lesions showing a histological picture of fibrosis and lymphoplasmacytic infiltrations with abundant IgG4 positive plasma cells at hitherto unreported symmetrical nasal locations. The symmetrical complex consisted of one central lesion in the anterior nasal septum and the two others in each of the lateral nasal walls. The lesions extended from the anterior part of the inferior concha into the vestibulum and caused severe nasal obstruction.

## 1. Introduction

IgG4-related disease was first associated with autoimmune pancreatitis. In 2003, it was recognized as a systemic condition [1] and lesions have been described in almost all organs, among them the nasal sinuses and the nasal septum [2, 3]. We present two cases with symmetrical pseudotumors at the nasal inlet causing severe nasal obstruction.

## 2. Case Reports

### 2.1. Case 1

A 34-year-old healthy Caucasian male complained of progressive nasal obstruction for several years. Clinically there was a severe narrowing of the nasal inlet due to three tumors in the soft tissue. One was located in the anterior part of the nasal septum; the other two were symmetrically located in both lateral nasal walls. The bulk of the lesions were in the vestibulum. A CT scan showed a thickening of the soft tissues at the nasal inlet without erosion of bony structures. The sinuses were clear. The tumors were removed surgically. At the first examination (2009) we were not aware of IgG4-related disease. The patient had no other respiratory symptoms or signs. Three years later there was a recurrence of all lesions dorsally involving the inferior conchae. The pseudotumors were resected relieving the obstruction. Serum IgG4 level was 1.630 g/L (normal value 0.030–2.010). The haematological values and the other serum IgG subclasses were within normal limits. The histology was identical to case number 2. During the ensuing nine months there has been no apparent recurrence of the manifestations.

### 2.2. Case 2

**Figure 1:**
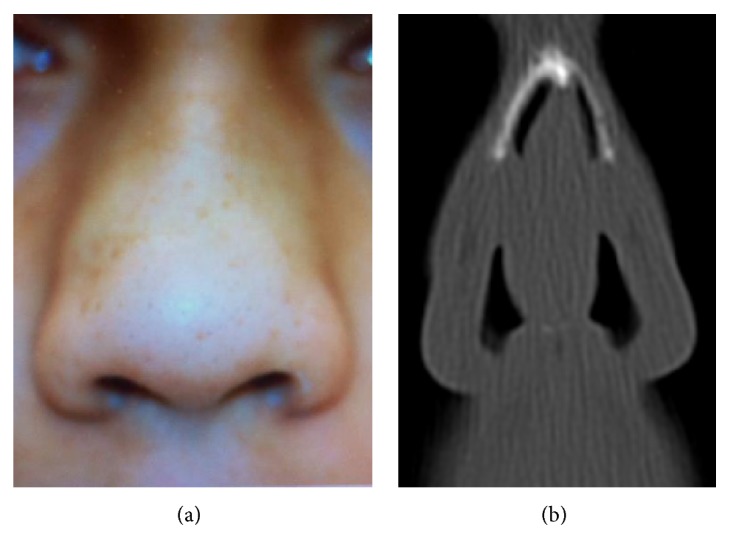
(a) Frontal view of the external nose. Note the swelling of the lateral walls. (b) A CT scan of the anterior nose. Note the wide nasal septum touching the swellings of the lateral pseudotumors which together cause a bulging of the lateral walls.

A 34-year-old woman originally from Sri Lanka complained of increasing nasal obstruction for two years. There was no history of trauma, infection, or surgery. Clinically, the soft tissues of the caudal end of the nasal septum, the inferior conchae, and the vestibuli were enlarged in size and created a swelling on the external nasal wall (Figure 1(a)). A CT scan showed normal sinuses and a wide caudal septum with a narrow airspace in the vestibulum and a widening of the lateral nasal walls (Figure 1(b)). There was no sign of bony erosion. A PET scan showed no other location of the disease. She had a history of acute pancreatitis eight years ago which spontaneously resolved. She has iron deficiency anaemia. The SR was 28 mm and the serum IgG4 level was 2.080 g/L. The other IgG subclasses, the white cell count, and ANCA were normal. Fibrous tumors were dissected free from the surrounding soft tissue in the caudal septum and in the anterior part of the inferior conchae. The nasal symptoms (obstruction and local pain) were not completely resolved and she was started on a low oral dose of corticosteroid therapy as she suffered from diabetes. This had limited effect, so supplementary treatment with rituximab was initiated. This eliminated the pain but had limited effect on the obstruction. The serum IgG4 level decreased to normal level (1.355 g/L). The histological description of the biopsies is given below.

## 3. Histology

**Figure 2:**
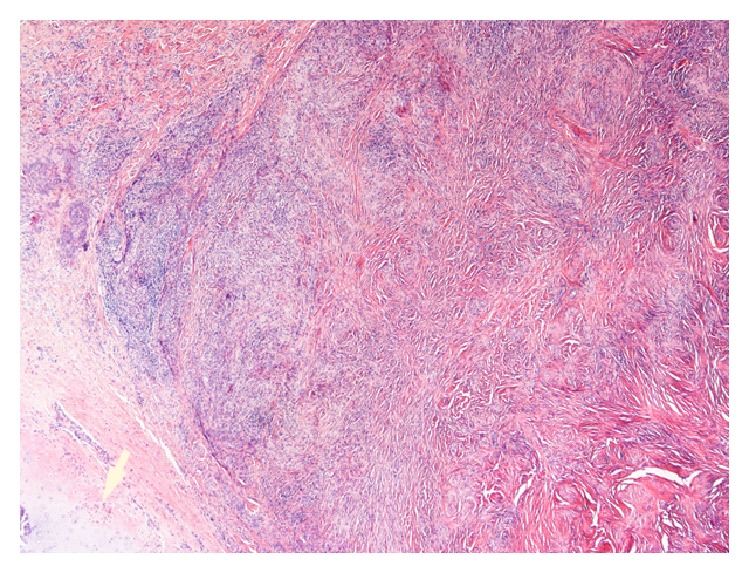
Massive fibrosis in a storiform pattern. Areas of accumulation of plasma cells. Destruction of normal mucosal structure. Cartilage from the nasal septum in the left bottom corner (arrow). HE ×20.

**Figure 3:**
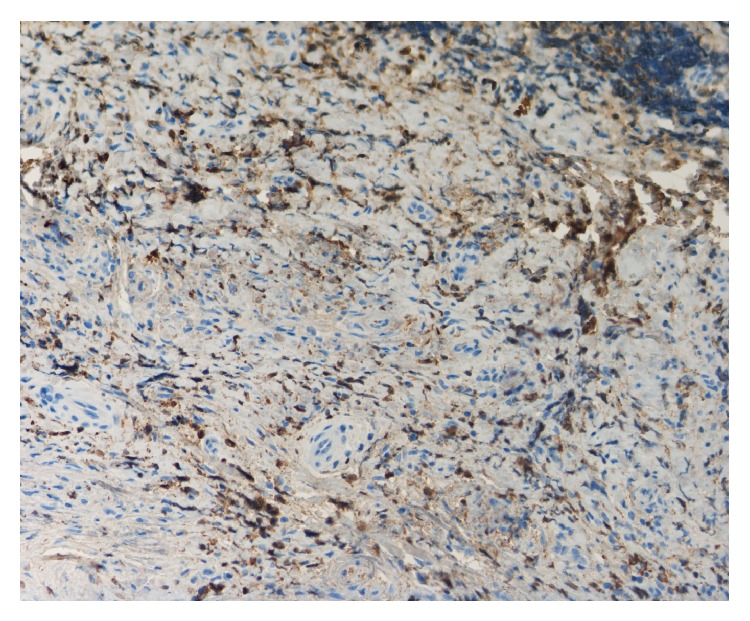
Immunohistochemical staining for total IgG in an area with inflammatory cells. The brown-coloured areas represent IgG. ×200.

**Figure 4:**
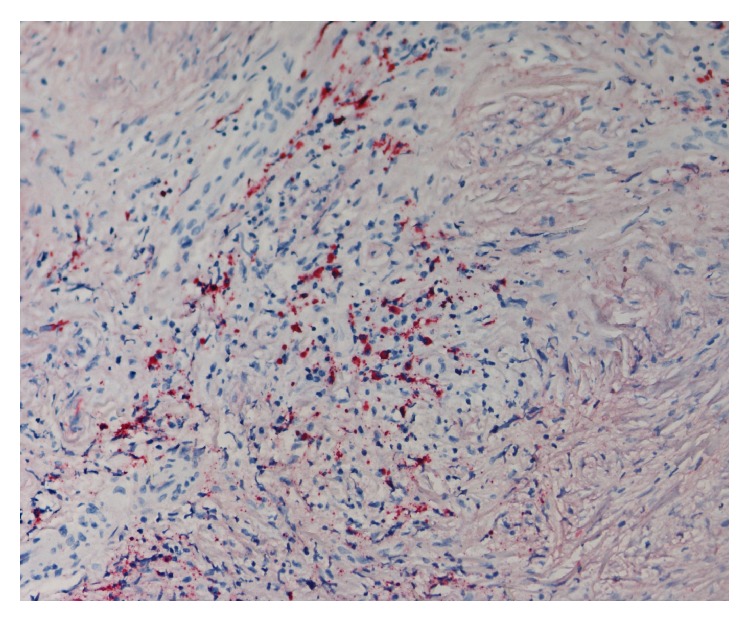
Immunohistochemical staining for IgG4 positive plasma cells. They were found in the same area as in Figure 3. They appear in red. ×200.

The biopsies from both patients showed the same histological features. The greater part of the tissue was fibrous with few cells (Figure 2). The pattern of this fibrous tissue in many places was storiform. In some areas there were accumulations of inflammatory cells. These were immunohistochemically stained for IgG (Figure 3) and IgG4 (Figure 4). Detection of IgG was made by using polyclonal rabbit anti-human antibody against IgG, specific for gamma-chains (Dako, Agilent Technologies). Mouse anti-human IgG4 antibody (Life Technologies) was used to detect IgG4. The number of IgG4+ plasma cells varied between 14 and 25 per high power field in the cell rich areas. The average ratio of IgG4+ to total IgG+ plasma cells in these areas was 7.7/10, well above the mandatory diagnostic ratio of 4/10.

## 4. Discussion

The diagnosis of IgG4-related disease is based on histology. The “Consensus Statement on the Pathology of IgG4-Related Disease” [4] lists three criteria as the major histopathological features of the disease. These are dense lymphoplasmacytic infiltration with an increase of IgG4 positive plasma cells, fibrosis with areas having a storiform pattern, and obliterative phlebitis. Two are required for the diagnosis. Fibrosis with a storiform pattern and plasma cell infiltrates with abundant IgG4 positive plasma cells were present in both our patients, thereby fulfilling the criteria for the diagnosis. The serum IgG4 level is often increased, but this is not diagnostic. It may be normal in up to 40% of patients [5]. Case 1 had a normal serum level of IgG4, whereas in case 2 it was slightly elevated.

IgG4-related pseudotumors have been reported in the nose and the sinuses. One was located in a maxillary sinus causing destruction of the bony sinus wall [2] and another in an ethmoidal sinus [6]. Multilocular occurrence has also been described. One patient had a lesion in a maxillary sinus and the nasal septum [3], another in both the maxillary and an ethmoidal sinus on the same side [7], and a third in a paranasal sinus and the nasal cavity [8]. All have been verified by histology according to the criteria described above. However, none of these three had the symmetrical lesion locations seen in our cases. We have not found any common traits between our patients besides the location of the disease. We cannot offer any explanation for this occurrence nor for the similarity of the location in two such different people. The diagnosis of the disease in the nasal area is difficult if histology is not performed. The age of our patients is markedly lower than in other publications of the disease, both in those with and without nasal manifestations [1–3, 5–7]. This may be explained by the location of the lesions. Obstruction at the narrowest part of the respiratory tract is bound to give symptoms at an early stage, whereas a lesion in other areas may go undetected for a long time.

IgG4-related autoimmune pancreatitis and Mikulicz disease have been associated with chronic rhinosinusitis (10 out of 31 patients) [8]. There was a high incidence of allergy among the rhinosinusitis patients. Biopsies from the nasal mucosa in those with chronic rhinosinusitis revealed inflammatory changes with numerous IgG4 positive plasma cells. The histology was indistinguishable from patients with chronic rhinosinusitis without IgG4-related disease. The histology showed no sign of fibrosis or phlebitis in either case, and the finding is inconsistent with the criteria used for the pseudotumors. The authors conclude, however, that chronic rhinosinusitis may be a manifestation of IgG4-related disease. The association between chronic rhinosinusitis and IgG4-related disease might be related to the high incidence of allergy, as an association between allergy and IgG4-related disease has been found in another study [9].

Nasal crusting and nasal obstruction were found in 10 of 23 patients with IgG4-related disease in other organs [10]. There was a significantly higher concentration of IgG4 positive plasma cells in the patients compared with controls. There was, however, no statistical difference between patients with or without nasal symptoms. There was no fibrosis or phlebitis in these biopsies.

IgG4 has been implicated in nasal disease. An increase in the number of IgG4 positive plasma cells without fibrosis or phlebitis has been found in the nasal mucosa in chronic rhinosinusitis both with and without IgG4-related disease in other organs. This increase, however, was not consistently present in the patients with nasal symptoms. Histology did not distinguish between rhinosinusitis with and rhinosinusitis without an increased concentration of IgG4 positive plasma cells. This is in strong contrast to the IgG4-related pseudotumors which exhibit specific histologic findings. Our patients had pseudotumors with characteristic criteria but no signs of allergy or chronic rhinosinusitis. The disease manifested itself with tumors arranged in a symmetrical pattern which resulted in nasal obstruction at a relatively early age.

These findings highlight the need for otorhinolaryngologists to be aware of the symmetrical nasal manifestations of IgG4-related pseudotumors. Given that the lesions recurred within three years in the first patient, surgery may not be the only therapeutic solution. One should be prepared to provide corticosteroids, particularly if the lesions recur.
